# Should All Patients Trial Subcutaneous Methotrexate Prior to Commencing Biologic Therapy? A Real World Study

**DOI:** 10.31138/mjr.140423.sat

**Published:** 2023-09-04

**Authors:** Anem Mirza, Muhammad K. Nisar

**Affiliations:** Department of Rheumatology, Luton & Dunstable University Hospital NHSFT, Luton, United Kingdom

**Keywords:** methotrexate, rheumatoid arthritis, psoriatic arthritis, biologics

## Abstract

**Introduction:**

Methotrexate (MTX) is the bed rock of inflammatory arthritis management. However, intolerance is a limiting factor for drug optimisation and retention. There is data to suggest subcutaneous (SC) MTX is better tolerated. It is less clear whether this strategy is effective in those where the oral preparation is inefficacious and its potential to avoid escalation to biologic therapy.

**Objectives:**

To analyse the reasons for switching to SC MTX in a real-world setting, clinical outcomes achieved and proportion requiring biologic prescription.

**Materials and Methods:**

A retrospective survey of patients prescribed SC MTX in a university teaching hospital identified 352 patients. 298 switched from oral to SC MTX- 164 stopped oral MTX due to side effects, 134 stopped due to inefficacy, and 54 started SC MTX as first line therapy. 103 patients progressed to biologic therapy. Rheumatoid arthritis (RA): DAS-28 improved from a mean of 4.06 (0.63–8.06) to 2.83 (0.14–7.32) following the switch (p<0.0001). Psoriatic arthritis (PsA): total joint count improved from a mean of 7 (0–42) to 2 (0–25) (p<0.0001). Swollen joint count improved from a mean of 2 (0–26) to 1 (0–6) (p=0.09).

**Discussion:**

SC MTX is an effective solution for RA and PsA, irrespective of whether oral MTX is inefficacious or intolerable. Where oral MTX was ineffective, a switch to SC achieved low disease activity despite multi-morbidity, long disease course and protracted oral MTX exposure. This intervention prevented over two-thirds of patients requiring biologics. SC MTX is a durable strategy with excellent disease outcomes and substantial economic benefits.

## INTRODUCTION

Rheumatoid arthritis (RA) remains a common rheumatological condition, affecting 1% of the population.^1^ Several bodies including National Institute for Health and Care Excellence (NICE) guidelines advocate a prompt diagnosis and a treat to target strategy with an aim of disease remission or low disease activity if remission is not possible.^2^ Conventional disease-modifying antirheumatic drugs (cDMARDs) have revolutionised the treatment of RA and many other inflammatory diseases, including psoriatic arthritis (PsA), and play a pivotal role in refining outcomes, improving prognosis, and achieving a better quality of life.^3^ In addition to NICE, both the American College of Rheumatology (ACR)^4^ and the European League Against Rheumatism (EULAR)^5^ guidelines recommend methotrexate (MTX) as an effective first line agent in the treatment of RA. If MTX is ineffective, other cDMARDs can be trialled, or advanced therapies, such as biologic agents.^2,4,5^ The endorsement of MTX by several different guidelines across the international community attests to its usefulness and cost effectiveness. In addition, oral MTX is easy to administer to patients.^2,4,5^

However, oral MTX has long been associated with side effects, in particular with gastrointestinal (GI) intolerance, which frequently limits dose escalation to an optimum dose and adherence with treatment, with up to 30% of patients in one retrospective study stopping oral MTX due to GI side effects.^6^

Subcutaneous (SC) MTX is a useful alternative, albeit more expensive than oral MTX.^7^ It is better tolerated, more efficacious (likely due to its parenteral absorption), relatively easy to administer and economical compared to advanced therapies including biosimilars. Indeed, the successful use of SC MTX following oral MTX related GI intolerance is well documented.^8^

Whilst it is well established that SC MTX is an effective alternative to oral MTX where GI intolerance or other side effects prove to be a barrier, the real-world evidence for its utility is lacking in those for whom oral MTX is ineffective.^9^ This is very relevant in everyday clinical practice as the introduction of SC preparation in the therapy ladder, following oral treatment inefficacy, ahead of more expensive advanced therapy, such as biologics or targeted synthetic (tsDMARDs), could achieve major financial savings for healthcare. Additionally, it could avoid unnecessary biologic monotherapy for instances where escalation is still required.

## OBJECTIVES

The purpose of this study was to analyse all patients at our unit with either a diagnosis of RA or PsA (an under looked area) prescribed SC MTX and to investigate the following:

- Reasons for SC MTX initiation
- Clinical outcomes as measured by disease activity score- 28 (DAS-28) for RA and tender joint count (TJC) and swollen joint count (SJC) for PsA for both oral and SC preparations of MTX
- Proportion of patients progressing to advanced therapy (biologics)

## MATERIALS AND METHODS

### Ethical approval

All procedures performed in studies involving human participants were in accordance with the ethical standards of the institutional and/or national research committee and with the 1964 Helsinki declaration and its later amendments or comparable ethical standards. A retrospective survey of all patients prescribed SC MTX at our large university teaching hospital between 1983 and April 2019 was performed (Institutional approval no 9/2017-18/Medicine/Rheumatology). This hospital serves a catchment area of 350,000 people with high levels of deprivation. 40% of the service users are from minority ethnic backgrounds.

All patients with RA and PsA follow treatment to target in a consultant led, nurse delivered pathway which involves prescription of oral MTX within two weeks of diagnosis and rapid escalation to 20–25mg/week over subsequent six weeks. At 12 weeks review (or earlier), if the treatment target remains unachieved or adverse events encountered, effectively disallowing dose escalation or oral MTX cessation, all patients are offered the switch to SC MTX aiming for a 20–25mg/week dosing regimen.

A large departmental clinical database was interrogated for all patients prescribed SC MTX, including full electronic health records with details such as co-morbidities, drugs, and disease management. Analysis of the following was performed:

- Demographics (age, ethnicity, gender, and comorbidities)
- Full drug history including polypharmacy.
- Duration of treatment (for oral MTX, SC MTX, biologics)
- Reasons for SC MTX initiation
- Clinical outcomes (as measured by DAS-28 for RA and TJC/SJC for PsA)
- Impact on biologic prescription

352 patients were identified who were prescribed SC MTX in the aforementioned timeframe.

These patients were assigned to one of three groups:

- Group 1: patients who had switched from oral MTX to SC due to inefficacy of oral MTX.
- Group 2: patients who switched from oral to SC MTX due to adverse effects.
- Group 3: patients who were started on SC MTX as first line therapy (did not have oral MTX).

Each group was further analysed based on their diagnosis.

Inefficacy for RA was defined as a DAS-28 of greater than 3.2 and more than three tender or swollen joints for PsA.

Adverse events were divided into four main domains, including GI disturbance, constitutional symptoms, abnormal liver function tests (LFT) and miscellaneous effects.

Statistical analysis was conducted using IBM SPSS Statistics 23 software and Epi Info version 7.0 (CDC Atlanta USA). Disease outcomes were compared using the two-tailed student t test. Change in disease activity upon switching from oral to SC MTX, and current disease activity on SC MTX was analysed. A p value of less than 0.05 was predefined as being statistically significant.

## RESULTS

### Demographics

A total of 352 patients prescribed SC MTX between 1983 and April 2019 were included in the study.

All patients with RA and PsA fulfilled 2010 EULAR/ACR RA and CASPAR criteria, respectively.

The mean age of the cohort was 54 years (3–87). 247 (70%) were women and 105 were men (30%). 260 (74%) were Caucasian, 64 (18%) Asian, 21 (6%) Afro-Caribbean, and 7 were of other ethnicity (2%). 284 patients had RA (81%) and 68 patients had PsA (19%). Median disease duration was 53 months (11–324) with a mean of three comorbidities (0–11).

The 352 patients were split into three groups as above. Each group was further divided into RA and PsA. The demographics are summarised in **Table 1**.

**Table 1.** Demographics.

	**Inefficacy Group**	**Adverse Events Group**	**SC MTX First Line Group**
**n**	134	164	54
**Disease**			
RA (n)	112	133	39
PsA (n)	22	31	15
**Gender**			
RA	M31, F81	M34, F99	M12, F27
PsA	M9, F13	M15, F16	M4, F11
**Mean Age (years)**			
RA	58 (17–87)	55 (22–84)	45 (3–75)
PsA	48 (20–75)	49 (25–82)	48 (5–77)
**Ethnicity: RA**			
White	87	88	29
Asian	20	26	8
Afro-Caribbean	4	16	0
Other	1	3	2
**Ethnicity: PsA**			
White	17	26	13
Asian	4	5	1
Afro-Caribbean	1	0	0
Other	0	0	1
**Drugs (RA)**			
*MTX only*	41	51	20
*Combination cDMARDs:*	28	54	10
MTX +Hydroxychloroquine	16	45	8
MTX + Sulfasazaline	2	3	0
MTX+Hydroxychloroquine+ Sulfasalazine	8	5	0
MTX+Hydroxychloroquine+Leflunomide	1	1	0
MTX + Leflunomide	0	0	1
MTX +Azathioprine	1	0	1
*Biologics*	43	28	9
**Drugs (PsA)**			
*MTX only*	12	16	9
*Combination cDMARDs:*	3	5	0
MTX +Hydroxychloroquine	0	0	0
MTX + Sulfasazaline	1	2	0
MTX+Hydroxychloroquine+ Sulfasalazine	1	2	0
MTX+Hydroxychloroquine+Leflunomide	0	0	0
MTX + Leflunomide	1	1	0
MTX +Azathioprine	0	0	0
*Biologics*	7	10	6

M: Male; F: Female; RA: Rheumatoid arthritis; PsA: Psoriatic arthritis; SC MTX: Subcutaneous methotrexate; cDMARD: Conventional disease modifying antirheumatic drugs. The mean age of the cohort was 54 years, 70% were women and 74% were Caucasian. 284 patients had RA (81%) and 68 patients had PsA (19%). 298 (85%) had switched from oral to SC MTX. The duration of oral MTX prior to switching was a mean of 26 months. 164 (47%) stopped oral MTX due to side effects, 134 (38%) patients switched due to inefficacy of oral MTX, and 54 patients (15%) started SC MTX as first line therapy. 103 (29%) patients progressed to biologic therapy.

Of the total (352) patients, 298 (85%) had switched from oral to SC MTX. The duration of oral MTX prior to switching was a mean of 26 months (0.25–167 months). 164 (47%) stopped oral MTX due to side effects, 134 (38%) patients switched from oral to SC MTX due to inefficacy of oral MTX, and 54 patients (15%) started SC MTX as first line therapy. Follow up period for SC MTX ranged from 2 to 132 months (mean 29 months) until the data cut-off date of April 2019. 103 (29%) patients progressed to biologic therapy.

### Overall disease outcome

Amongst the RA patients, the mean DAS-28 before commencing oral MTX was 4.63 (1.25- 7.59). DAS-28 at switch to SC MTX was 4.06 (0.63- 8.06). Final DAS-28 on current treatment was 2.83 (0.14–7.32).

PsA patients had a mean of 8 TJC (0–48) and 4 SJC (0–20) prior to the initiation of oral MTX and 7 TJC (0–42) and 2 SJC (0–26) at switching to SC MTX. Final TJC and SJC on current treatment was 2 (0–25) and 1 (0–6) respectively.

54 patients who were prescribed SC MTX as first line therapy were excluded from this analysis.

A summary of overall disease outcomes can be found in **Table 2**.

**Table 2.** Clinical outcomes pre- and post-switch to SC MTX.

	**Pre oral MTX**	**At switch to SC MTX**	**Post SC MTX**	**p value**
**RA n= 245**				
**DAS-28**				
Minimum	1.25	0.63	0.14	
Maximum	7.59	8.06	7.32	
Median	4.61	4.16	2.59	
Mean	4.63	4.06	2.83	p<0.0001
**PsA n= 53**				
**TJC**				
Minimum	0	0	0	
Maximum	48	42	25	
Median	6	5	0	
Mean	8	7	2	p<0.0001
**SJC**				
Minimum	0	0	0	
Maximum	20	26	6	
Median	2	1	0	
Mean	4	2	1	p=0.09

RA: Rheumatoid arthritis; DAS-28: Disease activity score-28; PsA: Psoriatic arthritis; SC: Subcutaneous; MTX: Methotrexate; TJC: Total Joint count; SJC: Swollen Joint Count.

RA: mean DAS-28 before commencing oral MTX was 4.63, DAS-28 at switch to SC MTX was 4.06 and final DAS-28 on current treatment was 2.83, p<0.0001

PsA: mean of 8 TJC and 4 SJC prior to oral MTX and 7 TJC and 2 SJC at switching to SC MTX. Final TJC and SJC on current treatment was 2 and 1 respectively. Reduction in TJC was statistically significant (p<0.0001) but reduction in SJC was not (p=0.09)

54 patients prescribed SC MTX as first line therapy were excluded from analysis.

Amongst RA patients, a statistically significant improvement was found in DAS-28 upon switching from oral MTX to SC MTX (p<0.0001). Similarly, a statistically significant improvement was achieved in PsA patients with respect to TJC pre- and post-SC MTX (p<0.0001) though SJC improvement was not significant (p=0.09).

### Adverse events with oral MTX

**Figure 1.**
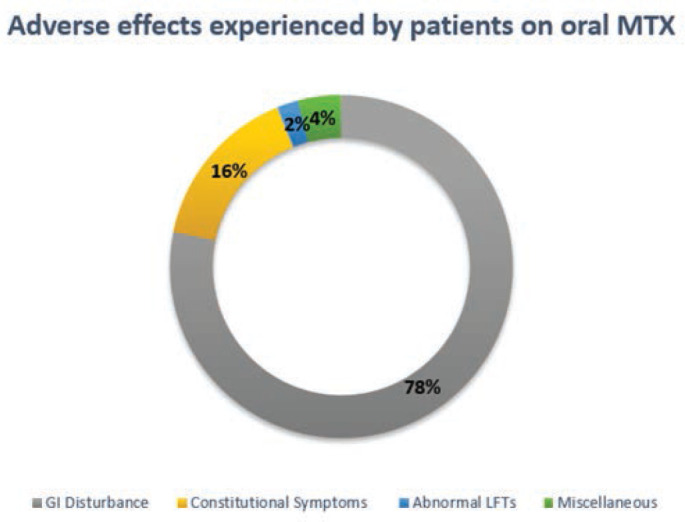
Adverse events with oral MTX (RA and PsA).

164 patients found oral MTX to produce adverse effects. 155 patients experienced GI intolerance, 31 patients had constitutional symptoms (of these patients, 15 complained of fatigue, 6 of headache, 4 of hair loss and 6 of mouth ulcers). Abnormal LFTs were observed in 4 patients. Miscellaneous symptoms were reported by 8 patients (these included 2 patients complaining of mood disturbance, 2 patients finding adverse effects troubling and choosing to switch to SC MTX, 1 patient reporting dizziness, 1 complaining of rash, 1 of ocular symptoms, and 1 who had an accidental overdose of oral MTX which necessitated a switch to SC MTX [**Figure 1**]).

155 patients experienced GI intolerance, 31 patients had constitutional symptoms (of these patients, 15 complained of fatigue, 6 of headache, 4 of hair loss and 6 of mouth ulcers). Abnormal LFTs were observed in 4 patients. Miscellaneous symptoms were reported by 8 patients (2 patients complaining of mood disturbance, 2 finding adverse effects troubling and choosing to switch to SC MTX, 1 reporting dizziness, 1 complaining of rash, 1 of ocular symptoms, and 1 who had an accidental overdose of oral MTX which necessitated a switch to SC MTX).

### Subgroup Analysis

#### RA

Amongst RA patients, DAS-28 improved from a mean of 4.06 (0.63–8.06) to 2.83 (0.14–7.32) following the switch from oral MTX to SC MTX.

In the RA inefficacy group (n=112), DAS-28 improved from a mean of 4.22 (range 1.20–8.06) pre-SC MTX to 3.02 (range 0.28–7.32) [p< 0.0001] following the switch. 43 (38%) ultimately progressed to biologics in this cohort. In the RA adverse events group (n=133), DAS-28 improved from a mean of 3.75 (range 0.63–7.15) to 2.64 (0.14–6.88) [p< 0.0001]. 28 (21%) ultimately progressed to biologics. In the SC MTX first line group, for RA (n=39), DAS-28 was not analysed as the purpose of this study was to compare patients who switched from oral MTX to SC and the impact this had on their disease. Of this group, 9 (23%) patients progressed to biologics due to treatment failure with cDMARDs. 10 patients were on combination cDMARDs.

#### PsA

Amongst patients with PsA, TJC and SJC improved from a mean of 7 (0–42) and 2 (0–26) to 2 (0–25) and 1 (0–6) respectively.

In the inefficacy group for PsA (n=22), TJC improved from a mean of 8 (0–28) to 3 (0–25) [p=0.0089] once SC MTX was instituted. SJC improved from mean of 3 (0–13) to 2 (0–6) [p= 0.1992] with SC MTX. 7 (32%) ultimately progressed to biologics.

In the adverse events group (n=31), TJC improved from a mean of 4 (0–22) to 1 (0–9) [p= 0.0070] following SC MTX prescription. SJC improved from a mean of 1 (0–8) to 0 (0–3) [p<0.0001] with SC MTX. 10 (32%) were prescribed biologics.

In the SC MTX first line group, for PsA (n=15), TJC/SJC were not analysed as the purpose of this study was to compare patients who switched from oral MTX to SC and the impact this had on their disease. Of this group, 6 (40%) patients progressed to biologics due to treatment failure with cDMARDs. None of these patients were on combination cDMARDs.

See **Table 3** for a summary of the subgroup analysis. There were 54 patients who were started on SC MTX as first line therapy. The reasons for this are outlined below in **Table 4**.

**Table 3.** Disease outcomes by subgroups.

	**Inefficacy Group**	**P value on switching from oral to SC MTX**	**Adverse Events Group**	**P value on switching from oral to SC MTX**
**RA n=**	112	p<0.0001	133	p<0.0001
**Number progressing to biologics**	43		28	
**DAS-28 Mean**				
Pre-Oral MTX	4.75 (1.89- 7.59)		4.54 (1.25-7.49)	
Pre-SC MTX	4.22 (1.20- 8.06)		3.75 (0.63- 7.15)	
Current	3.02 (0.28- 7.32)		2.64 (0.14- 6.88)	
**PsA n=**	22	TJC p=0.0089 SJC P=0.1992	31	TJC p=0.0070 SJC p<0.0001
**Number progressing to biologics**	7		10	
**TJC Mean**				
Pre-Oral MTX	10 (1–48)		7 (1–22)	
Pre-SC MTX	8 (0–28)		4 (0–22)	
Current	3 (0–25)		1 (0–9)	
**SJC Mean**				
Pre-Oral MTX	4 (0–20)		4 (0–14)	
Pre-SC MTX	3 (0–13)		1 (0–8)	
Current	2 (0–6)		0 (0–3)	

RA: Rheumatoid arthritis; DAS-28: Disease activity score-28; PsA: Psoriatic arthritis; SC: Subcutaneous; MTX: Methotrexate; TJC: Total Joint count; SJC: Swollen Joint Count.

RA inefficacy, DAS-28 improved from mean of 4.22 pre-SC MTX to 3.02 [p< 0.0001] following the switch. 38% ultimately progressed to biologics.

RA adverse events, DAS-28 improved from mean of 3.75 to 2.64 [p< 0.0001]. 21% progressed to biologics.

PsA inefficacy, TJC improved from mean of 8 to 3. SJC improved from mean of 3 to 2 [p= 0.1992] with SC MTX. 32% progressed to biologics.

PsA adverse events, TJC improved from mean of 4 to 1 [p= 0.0070]. SJC improved from a mean of 1 to 0 [p<0.0001]. 32% were prescribed biologics.

**Table 4.** Reasons for starting SC MTX as first line therapy (RA and PsA).

**Reason for Starting SC MTX**	**n=**
As per local protocol for children with rheumatological disease	8
Patient choice/anxiety over side effects of oral MTX	17
Higher efficacy/better absorption of SC MTX	20
Pre-existing prescription	9

RA: Rheumatoid arthritis; PsA: Psoriatic arthritis; SC MTX: Subcutaneous Methotrexate.

NB: where better absorption of SC MTX was a primary motivator for starting SC MTX, these patients either had a high body mass index (BMI) or concurrent diagnosis of inflammatory bowel disease (IBD).

## DISCUSSION

This large real-world study in a diverse teaching hospital setting confirms that switching to SC MTX is an effective option for patients with inflammatory arthritis, irrespective of whether the oral preparation was inefficacious or poorly tolerated. Our study provides unique data for PsA patients who achieve similar outcomes to the well published RA cohort. By switching to SC MTX from oral MTX, three-quarters of patients in this study were able to avoid a further escalation to biologics and this has major financial implications for healthcare systems worldwide. Our data confirms significant improvement in RA for patients who switched from oral to SC MTX preparation thereby providing confidence to clinicians that SC MTX is a cost-effective step in the therapy ladder prior to considering higher cost advanced therapies. This concurs with published evidence demonstrating that switching from oral to SC route is in line with the treat-to-target strategy and with known pharmacokinetics of injectable MTX. ^10–13^

Oral MTX bioavailability is reported to be between 30% to 70%^10, 11^ and reaches a plateau with a single oral dose over 15 mg^12^ suggesting an absorption limitation.^13^ SC route increases MTX bioavailability, regardless of the dose. ^12^ Switching from oral to SC MTX may improve clinical response in RA patients with inadequate response ^12, 14–16^ and prevent gastrointestinal side effects. ^17^ Furthermore, SC route has been previously shown to improve adherence to MTX. ^18–19^

Interestingly, our study also demonstrates the utility of this strategy in PsA. The use of SC MTX was found to improve the TJC in a statistically significant manner, but not the SJC. Reason for this disparity in TJC and SJC might be the small sample size (n= 53) however it may also reflect the fact that PsA is a heterogenous disorder, and its clinical course is distinct to RA.^20^ Despite these findings, SC MTX was successful in preventing the need to escalate to biologics for most of the patients, thus it could be argued that for most patients, SC MTX is useful at controlling the symptoms of PsA.

In our study, oral MTX was ineffective in 45% of patients. This has been shown previously with one observational study reporting 37% of patients with oral MTX experienced inefficacy.^21^ We also found that over half of the patients in this study experienced side effects with oral MTX with the vast majority experiencing GI intolerance. The adverse effects of oral MTX therapy are well established and have implications for adherence, limited dose titration to an optimum or continuation of treatment with MTX. ^18–19^

In about one-fifth of patients, SC MTX was prescribed first line. Whilst SC MTX is commonly employed for the treatment of juvenile idiopathic arthritis, ^22^ our study shows its utility in other clinical settings and confirms that shared decision making with MDT input makes it an effective approach; although, it should be noted that approximately one third of patients who were prescribed SC MTX in the first instance still did require escalation to advanced treatments due to treatment failure.

In the UK, NICE guidelines recommend a minimum of two cDMARDs for at least six months before considering escalation to advanced therapies.^23^ Biologics remain an expensive option to treat inflammatory conditions.^24^

Using SC MTX instead of biologics after oral MTX discontinuation, either due to adverse effects or inefficacy, means that up to £7000 can be saved in the first year of treatment in comparison to using biologics.^23^ By adding SC MTX in the treatment algorithm, we avoided biologics, for under a third of our patients.

Similar cost saving effects with the usage of SC MTX versus biologics have been found in the USA with biologics being associated with a cost that is three- four times higher compared to oral or SC MTX. In addition, the use of SC MTX has been found to delay the need to start biologics by a mean of 706 days. ^25^

In our study, we also found that the use of SC MTX (switching from oral MTX to SC instead of direct to biologics) was able to delay the need to start biologics by a mean of 842 days. Of the smaller group of patients (n=54) who started SC MTX in the first instance, there was a delay in starting biologics by a mean of 732 days. We have demonstrated that the use of SC MTX not only significantly reduces the rate of progression to advanced therapies, but it can also avoid them altogether. This has major implications for healthcare systems internationally as cost-effective prescribing is of the utmost priority**.** What our study adds to the existing literature is the finding that by switching from oral MTX to SC MTX it is possible to achieve disease remission or low disease activity for patients who experience inefficacy or adverse effects of oral MTX. These factors may favour starting patients directly on SC MTX, instead of starting PO MTX and then switching to SC MTX (if required). However, cost will be an important consideration with such an approach.

The retrospective nature of this single urban centre study has several limitations. Arguably, the results may not be generalisable to all clinical settings. The reimbursement criteria for advanced therapies are also different around the world, thus the financial benefits or implications of delaying the prescription of advanced therapies may not be relevant to some countries. Furthermore, our study period of 36 years, relatively large number of patients including those with PsA, long follow up and availability of detailed drug and disease parameters counteract some of these constraints.

## CONCLUSION

SC MTX should be considered as a treatment option for people with RA or PsA who are unable to continue with oral MTX. SC MTX is an effective treatment option for both inefficacy and intolerance of oral preparation and can delay or even circumvent the need for high-cost drugs.

## KEY POINTS

- SC MTX is a highly effective treatment for RA and PsA.
- Usage of SC MTX in these conditions can delay or reduce the need to escalate to advanced therapy such as biologics.
- SC MTX is a cost-effective treatment compared to biologics and should be considered in people with inflammatory arthritis who are either intolerant to oral MTX or it is inefficacious.

## DECLARATIONS

Previous abstract publications (work presented as a poster at European Congress of Rheumatology): Mirza A, Nisar M.K. Should all patients trial subcutaneous methotrexate prior to commencing biologic therapy- a real world study [abstract]. Annals of The Rheumatic Diseases 2021; 80 (Supplement 1): 333.
